# The Effects of Leg Preference on Transient Characteristics of Body Sway During Single-Leg Stance: A Cross-Sectional Study

**DOI:** 10.3389/fnhum.2020.617222

**Published:** 2021-01-11

**Authors:** Žiga Kozinc, Nejc Šarabon

**Affiliations:** ^1^Faculty of Health Sciences, University of Primorska, Izola, Slovenia; ^2^Andrej Marušič Institute, University of Primorska, Koper, Slovenia; ^3^Human Health Department, InnoRenew CoE, Izola, Slovenia; ^4^S2P, Science to Practice, Ltd., Laboratory for Motor Control and Motor Behavior, Ljubljana, Slovenia

**Keywords:** sensory integration, sensory reweighing, leg preference, transient behavior, body sway, postural control

## Abstract

Instrumented assessments of quiet-stance postural control typically involve recording and analyzing of body sway signal, most often the center of pressure (CoP) movement. It has been recently suggested that transient characteristics of body sway may offer additional information regarding postural control. In this study, we explored the relationship between whole-trial estimates of body sway (CoP velocity, amplitude, and frequency) and corresponding transient behavior indexes, as well as the effects of leg preference. A total of 705 healthy young athletes performed 30 s single-leg body sway trials for both legs. It was found that the transient characteristics of the body sway (expressed as relative differences between individual time intervals within the trial) are in negligible or weak correlation (*r* ≤ 0.26) with the corresponding variables, averaged across the whole trial. All CoP variables showed transient characteristics, reflected in statistically significant decrease (CoP velocity and amplitude) or increase (CoP frequency) throughout the trial. The preferred leg showed smaller body sway; however, the effect sizes were very small. Moreover, differences between the legs were also noted in terms of transient characteristics of body sway. In particular, the preferred leg showed earlier reduction in anterior–posterior body sway and larger reduction in medial–lateral body sway. Further studies should focus on examining the clinical utility of indexes of transient behavior of body sway, for instance, their sensitivity to aging-related changes and risk of falling.

## Introduction

The examination of quiet-stance postural control by assessing body sway characteristics during quiet standing is routinely used in research and practice in several fields related to human health and human movement (Piirtola and Era, 2006; Ku et al., 2014; Yen et al., 2016; Roman-Liu, 2018; Kozinc et al., 2020). For instance, variables related to center of pressure (CoP) movement may be used to determine the risk of falling in older adults (Piirtola and Era, 2006; Kozinc et al., 2020) and to assess balance deficits in Parkinson’s disease patients (Gera et al., 2016; Feller et al., 2019). Postural balance is achieved by integrating sensory information from visual, vestibular, and somatosensory systems; planning; and execution of appropriate neuromuscular responses (Peterka, 2002, 2018). The most common assessment of body sway is done by recording CoP movement during quiet standing. Several studies have also explored the effects of elimination (e.g., closing the eyes to eliminate the visual system) or manipulation (e.g., using compliant base of support to manipulate somatosensory information) of one or more of the systems (Assländer and Peterka, 2016; Gera et al., 2016; Peterka, 2018; Appiah-Kubi and Wright, 2019; Feller et al., 2019). Such investigations contribute to the understating of the underlying mechanisms of postural control (Peterka, 2002, 2018; Assländer and Peterka, 2016) and may also provide additional clinical utility for recognition of postural balance deficits (Gera et al., 2016; Appiah-Kubi and Wright, 2019; Feller et al., 2019). For example, the ability to adapt quickly and efficiently to changes in sensory information inflow (i.e., the sensory reweighing) could play an important role in certain real-life situations (e.g., walking when lights are turned off, unexpected changes in supporting surface, etc.) (Peterka, 2018). Moreover, previous studies have also used the sensory reweighing analyses to show sport-specific sensory integration (Thalassinos et al., 2018). Specifically, it was shown that ballet dancers were destabilized more than soccer players when their Achilles tendons were subjected to vibration, which disturbs the somatosensory information. The ballet dancers also required more time to reweigh sensory information, which was believed to be a sport-specific adaptation characterized by heavier reliance on somatosensory information (Thalassinos et al., 2018).

It is clear that assessment procedures that extend beyond recording the CoP characteristics during quiet stance are an important addition, in both scientific and practical terms. However, the responses of body sway to changes in sensory conditions are still largely unexplored (Reed et al., 2020). For example, the “double-leg to a single-leg stance transition test” was proposed for clinical and laboratory assessment of postural stability (Wiesław Błaszczyk et al., 2020); however, its clinical utility is yet to be determined. Another promising area that has recently re-emerged is the assessment of transient characteristics of body sway. It has been argued that certain part of the information related to the characteristics of body sway is “lost” when averaging across the whole quiet-stance trials (e.g., 60 s of quiet standing) (Reed et al., 2020). In other words, as stressed by Reed et al. (2020), the whole-trial estimates may mask transient postural characteristics (i.e., an initial destabilized period that is followed by a transition to a more stable, quasi-steady state level). To address this question, the authors assessed transient body sway characteristics by calculating absolute differences among individual 5 s intervals during the 60 s trial. They reported no associations between the whole-trial estimates of CoP parameters and the absolute changes (in the corresponding CoP parameters) between the individual 5 s intervals (Reed et al., 2020). This indicated that the transient characteristics of body sway (i.e., the differences between the intervals within a longer trial) could represent additional information regarding the postural control. Previously, a potential clinical relevance of similar approaches has been reported for Parkinson’s disease (Brown et al., 2006) and diabetic neuropathy (Boucher et al., 1995); however, the transient body sway characteristics remain largely unexplored.

In this paper, we focus predominantly on the effects of limb preference on whole-trial estimates and transient characteristics of body sway. Previous studies have investigated the differences between the preferred and non-preferred limbs in terms of body sway variables (Hoffman et al., 1998; Matsuda et al., 2008; Barone, 2010; Alonso et al., 2011; Muehlbauer et al., 2014; Promsri et al., 2018); however, statistically significant differences between the limbs were scarcely reported (Barone, 2010; Promsri et al., 2018). It could be that the differences between the limbs are small and not easily detected with small sample sizes. Therefore, it remains unclear if leg preference is associated with quiet-stance body sway variables. Furthermore, it could be that different approaches to classification of the limb as preferred would yield different results. Typically, the preferred leg is determined as self-reported leg with which an individual would perform manipulative tasks, such as kicking a ball (Matsuda et al., 2008; Barone, 2010; Bishop et al., 2018). On the other hand, different approaches, such as choosing the preferred leg for single-leg jumping, are rarely used (Bishop et al., 2018) and have not been explored in connection with postural control. Moreover, to the best of our knowledge, no previous study has investigated inter-limb differences regarding the transient characteristics of body sway.

In view of the outlined ambiguities, we performed several exploratory analyses, using a previously collected database containing more than 700 participants, who all performed single-leg body sway assessments with open eyes. We chose single-leg standing balance because of its similarity with the movements in sports that require balancing on single-leg (e.g., dance and martial arts elements, soccer kicks, basketball pivoting and jumping, etc.). Moreover, because our sample presumably had very efficient postural control, parallel stance test would likely fail to detect any meaningful difference. On the other hand, we also opted not to include eyes closed condition with the single-leg stance, because the difficulty of such test could result in excessive noise in the data. Moreover, in contrast with Reed et al. (2020), we used slightly longer time intervals (10 s instead of 5 s). It has been noted that the reliability body sway assessment is decreased with lower trial durations (Carpenter et al., 2001); therefore, very small intervals used for transient characteristics calculation could result in larger errors. The first primary aim of the study was to assess the relationships between whole-trial estimates and transient characteristics of body sway. This is a preliminary step that shows whether the transient characteristics are independent from the whole-trial estimates and can thereby represent additional valuable information regarding individual’s postural control. Our second primary aim was to explore the effects of the leg preference on whole-trial estimates and transient characteristics of body sway. We compared two approaches to classification of the preferred limb. We hypothesized that the whole-trial estimates and transient characteristics of corresponding body sway variables will show negligible or small associations (Reed et al., 2020). We also expected that body sway will stabilize (i.e., decrease) throughout the trial, and that there will be no differences between the preferred and non-preferred legs (Hoffman et al., 1998; Matsuda et al., 2008; Barone, 2010; Alonso et al., 2011; Muehlbauer et al., 2014; Promsri et al., 2018).

## Materials and Methods

### Participants

The data were collected within a larger study that explored inter-limb asymmetries in different athletic populations. The study sample comprised 705 participants (508 males, 197 females) who were mostly junior and senior athletes from various sport disciplines [soccer = 209, basketball = 165, tennis = 109, long-distance running = 49, hip hop dancing = 34, martial arts (karate and ju-jitsu) = 34, and short-distance running = 28]. The remaining 77 participants were university students of physical education. The sample characteristics were as follows (mean ± standard deviation): age = 18.7 ± 6.9 years, body mass = 69.8 ± 12.8 kg, and body height = 177.8 ± 10.5 cm. The inclusion criteria were the absence of any musculoskeletal injuries within the last 3 months, as well as any neurological diseases and chronic non-communicable diseases. The study protocol was approved by the National Medical Ethics Committee (approval number: 0120-99/2018/5) and was compliant with the Helsinki Declaration. An additional consent was provided by legal guardians for all underage participants.

### Study Design, Tasks, and Procedures

The study was a single-visit cross-sectional experiment, conducted within a larger study. The body sway assessment was included as a part of an extended protocol that involved the assessment of passive range of motion, isometric lower limb strength, and vertical jumping ability. The body sway was assessed during single-leg stance on a force platform. The hip of the free leg was at 0°, and the thigh was parallel to the standing leg, whereas the knee was flexed at 90° and was not allowed to be touching the standing leg. The knee of the standing leg was extended with the instruction not to lock (i.e., hyperextend). The participants were instructed to look at a fixed point (black dot on a white background, at an approximately eye level and ∼4 m away from the participant). The hands had to be placed on the hips. Three 30 s repetitions were performed for each leg, with 60 s breaks between repetitions. For each repetition, the participants assumed the single-leg position, and the examiner started the acquisition after ∼1 s. Both legs were assessed and were alternated across repetitions, whereas the starting leg was randomized between participants. The position of the foot was marked on a force platform with a tape and was kept constant across repetitions.

### Determination of Leg Preference

Two approaches were used to determine the preferred and the non-preferred leg. In the first approach, the preferred leg was determined as “the leg you would use for single-leg jumps to achieve maximal jump height.” The second approach was based on the participant’s handedness, meaning that the preferred leg was matched with their preferred hand for writing and eating. It has been shown that this matches almost perfectly (in 96% cases) with the preferred leg for “kicking a ball” (Promsri et al., 2018). Therefore, a third approach, asking for the preferred leg for kicking a ball, was not used.

### Data Processing and Outcome Measures

The ground reaction force data were collected (sampling frequency: 1,000 Hz) by a force platform (model 9260AA; Kistler, Winterthur, Switzerland) and automatically low-pass filtered (Butterworth, 2nd order, 10 Hz) within the manufacturer’s MARS software (version 4.0; Kistler, Winterthur, Switzerland). The data were further automatically processed in the same software in order to obtain the outcome variables of interest. For all the outcome variables, the average of the three repetitions was used for further analyses. We obtained mean CoP velocity [total, anterior–posterior (AP), and medial–lateral (ML)], CoP amplitude (AP and ML), and CoP frequency. The CoP amplitude was defined as the average amount of the CoP sway in AP or ML direction, calculated as the common length of the trajectory of the COP sway only in the given direction, divided by the number of changes of movement direction. The CoP frequency was defined as the frequency of the oscillations of the CoP calculated as the number of peaks in AP or ML direction (i.e., changes in direction of CoP movement) divided by the measurement time. Firstly, we calculated the traditional whole-trial estimates (i.e., averaged CoP characteristics over the whole 30 s of the trial). In addition, we also calculated the relative differences between the 1st and the 2nd (DIF_21) and the 1st and the 3rd (DIF_31) 10 s time intervals within the whole trial. These relative differences were expressed as percentages (100% representing no change, >100% indicating an increase in time, <100% indicating a decrease in time).

### Statistical Analysis

Statistical analysis was done in SPSS (version 25.0; SPSS Inc., Chicago, IL, United States). Descriptive statistics were calculated and reported as mean ± standard deviation (tables) and as mean ± standard error of mean (figures). The normality of the data distribution was checked with Shapiro–Wilk tests (*p* ≤ 0.121). Within-participant variation among the repetitions was assessed by coefficient of variance (CV). A three-way analysis of variance (ANOVA) with two within-participant factors [i.e., leg (2) and time interval (3)] and one between-participant factors [sport discipline (9)] was conducted to explore how the leg preference and choice of a time interval affected the body sway and whether this behavior was different between groups of athletes. For comparison of the three time intervals and for comparisons of the legs at individual time intervals, the *post hoc t*-tests with Bonferroni correction were used. The effect sizes pertaining to ANOVA were expressed as partial eta-squared (η^2^) and interpreted as small (<0.13), medium (0.13–0.26), and large (>0.26) (Bakeman, 2005), whereas the effect sizes for *t*-tests were calculated as Cohen’s d (0.0–0.2—trivial, 0.2–0.6—moderate, 0.6–1.2—large, and >1.2—very large) (Bernards et al., 2017). Correlations between whole-trial outcome variables and corresponding transient characteristic variables (DIF_21 and DIF_31) were assessed by Pearson’s correlation coefficients and interpreted as negligible (<0.1), weak (0.1–0.4), moderate (0.4–0.7), strong (0.7–0.9), and very strong (>0.9) (Schober and Schwarte, 2018). For all analyses, the threshold for statistical significance was set at *p* < 0.05.

## Results

The first classification approach according to the leg preference for single-leg jumping resulted in 458 left legs and 247 right legs classified as preferred. On the other hand, according to handedness approach, 63 left legs and 643 right legs were classified as preferred.

Within-participant CVs were all below 10% for the whole-trial estimates (CV = 3.5–9.5%). For the individual time intervals, the CVs were mostly within 10% (CV = 5.1–9.4%), except for all of the CoP amplitude variables at different time intervals (CV = 11.7–17.6). Similarly, for DIF_21 and DIF_21, the CVs were below 10% for most variables (CV = 7.5–9.5), except for all CoP amplitude variables (CV = 12.5–19.9%).

### Correlations Among Whole-Trial Transient Characteristics of Body Sway

Correlations among whole-trial estimates and corresponding transient characteristics of body sway are summarized in Table 1. The analyses are shown only related to the classification of the preferred leg as the jumping leg. The results for the handedness-based preference were very similar (all *r* < 0.24). Generally, the correlation coefficients were trivial to small (−0.026 ≤ *r* ≤ 0.265). All correlations for the preferred leg were statistically significant (*p* < 0.01). On the other hand, only the correlations pertaining to CoP AP velocity and amplitude and CoP ML frequency were statistically significant (*p* < 0.05). Correlations were very similar (difference in *r* = 0.01–0.07) if individual groups of subjects were examined separately.

**TABLE 1:** Correlations among whole-trial estimates and corresponding transient characteristics of body sway.

Outcome variable	Preferred leg (jumping)		Non-preferred leg (jumping)	
	DIF_21	DIF_31	DIF_21	DIF_31
CoP velocity—total (mm/s)	0.201**	0.153**	0.052	0.043
CoP velocity—AP (mm/s)	0.265**	0.225**	0.121**	0.118**
CoP velocity—ML (mm/s)	0.131**	0.102**	–0.003	–0.026
CoP amplitude—AP (mm)	0.207**	0.209**	0.099**	0.095*
CoP amplitude—ML (mm)	0.109**	0.125**	0.004	0.011
CoP frequency—AP (Hz)	0.091*	0.096*	–0.024	0.051
CoP frequency—ML (Hz)	0.143**	0.170**	0.073	0.156**

*p < 0.05; **p < 0.01; CoP, center-of-pressure; AP, anterior–posterior; ML, medial–lateral; DIF_21, relative difference between the 1st and 2nd intervals within the whole trial; DIF_31, relative difference between the 1st and 3rd intervals within the whole trial.

### Effects of Leg Preference and Time Interval

For both approaches of classification of the preferred and non-preferred legs, in view of all CoP parameters, the ANOVA showed the absence of leg × time interval × group interactions (*p* = 0.455–0.987), as well as leg × group (*p* = 0.243–0.888) and time interval × group (*p* = 0.311–9.23). The main effect of group was statistically significant for all whole-trial- and interval-specific variables (*p* < 0.001), but not for DIF_21 and DIF_31 (*p* = 0.187–0.866). Because the group comparisons are not the focus of the study (and because all the above mentioned interactions were all statistically non-significant), we further treated the sample as one group in terms of assessing the effects of the leg, the time interval, as well as leg × time interval interactions.

When the legs were classified according to preference for single-leg jumping (Table 2), the effect of the leg (i.e., preferred vs. non-preferred) was statistically significant for velocity and amplitude variables (*p* = 0.001–0.028) with small effect sizes (η^2^ = 0.01–0.02), whereas there was no effect of the leg on the frequency variables (*p* = 0.411–574). The time interval had statistically significant effect on all outcome variables (*p* < 0.001), with effect sizes ranging from small to large (η^2^ = 0.05–0.42). No statistically significant interactions between leg and time interval were found (*p* = 0.165–0.705, η^2^ ≤ 0.01). Pairwise *post hoc t*-test showed that the differences were present between all three time intervals for all velocity and amplitude variables (all *p* < 0.001).

**TABLE 2:** Two-way analysis for variance for effects of leg and time interval on body sway outcome variables.

Preference—jumping	Leg			Time interval			Leg × time interval		
	*F*	*p*	η^2^	*F*	*p*	η^2^	*F*	*P*	η^2^
CoP velocity—total (mm/s)	9.19	0.003	0.01	268.7	0.000	0.28	2.01	0.134	0.01
CoP velocity—AP (mm/s)	11.19	0.001	0.02	66.87	0.000	0.10	1.27	0.280	0.01
CoP velocity—ML (mm/s)	4.84	0.028	0.01	511.1	0.000	0.42	1.80	0.165	0.01
CoP amplitude—AP (mm)	10.10	0.002	0.01	74.14	0.000	0.10	0.35	0.702	0.01
CoP amplitude—ML (mm)	6.97	0.008	0.01	357.4	0.000	0.34	0.34	0.705	0.00
CoP frequency—AP (Hz)	0.316	0.574	0.00	30.94	0.000	0.05	1.61	0.199	0.01
CoP frequency—ML (Hz)	0.676	0.411	0.01	85.55	0.000	0.11	1.58	0.204	0.01

**Preference—handedness**	**Leg**			**Time interval**			**Leg × time interval**		
	***F***	***p***	**η^2^**	***F***	***p***	**η^2^**	***F***	***P***	**η^2^**

CoP velocity—total (mm/s)	4.61	0.032	0.01	269.2	0.000	0.28	3.33	0.036	0.01
CoP velocity—AP (mm/s)	12.55	0.000	0.02	67.02	0.000	0.09	3.08	0.046	0.01
CoP velocity—ML (mm/s)	0.69	0.432	0.01	511.5	0.000	0.42	3.063	0.047	0.01
CoP amplitude—AP (mm)	14.4	0.000	0.02	74.38	0.000	0.10	3.629	0.027	0.01
CoP amplitude—ML (mm)	8.82	0.003	0.01	357.7	0.000	0.34	1.088	0.337	0.01
CoP frequency—AP (Hz)	0.46	0.496	0.01	59.05	0.000	0.08	0.741	0.477	0.01
CoP frequency—ML (Hz)	23.4	0.000	0.03	114.7	0.000	0.14	0.050	0.952	0.00

*CoP, center-of-pressure; AP, anterior–posterior; ML, medial–lateral.*

When the legs were classified according to the handedness (Table 2), the effects of time interval were similarly large (η^2^ = 0.08–0.42, all *p* < 0.001), with statistically significant and small (η^2^ = 0.01–0.03) effect of leg were present for most variables, except CoP ML velocity and CoP AP frequency (*p* = 0.432 and 0.496, respectively). Statistically significant leg × time interval interactions were found for all CoP velocity variables (*p* = 0.036–0.047) and CoP AP amplitude (*p* = 0.027), although the effect sizes were all small (η^2^ = 0.01).

**FIGURE 1:**
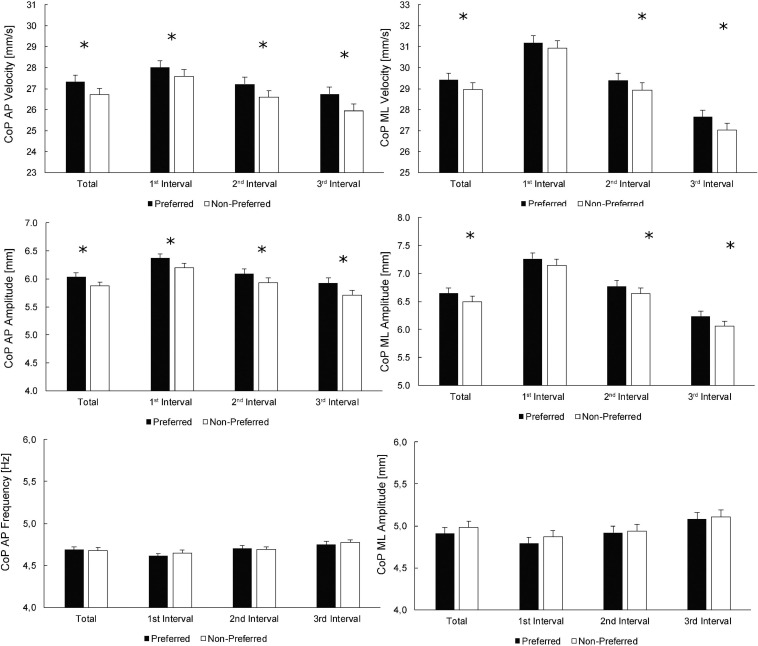
Comparisons between the preferred and non-preferred legs (according to the preference for single-leg jumping). Statistically significant differences between the legs for whole-trial (total; leftmost columns) values, as well as for individual time intervals, are denoted by asterisks. For main effects of leg and time intervals, see Table 2 (top half).

Figure 1 depicts the values for the preferred and non-preferred legs, according to the preference for single-leg jumping, for both the whole-trial estimates as well as the values at individual intervals. Statistically significant effects of the leg, assessed by pairwise *t*-tests, were found for all velocity and amplitude variables at all times, except for CoP velocity ML and CoP amplitude ML at the 1st interval. Similarly, to the main effect of the leg within the ANOVA, the effect sizes for statistically significant differences between the legs were small (Cohen’s *d* = 0.03–0.09). There were no statistically significant effects of the leg preference on CoP frequency variables (*p* ≥ 0.322).

**FIGURE 2:**
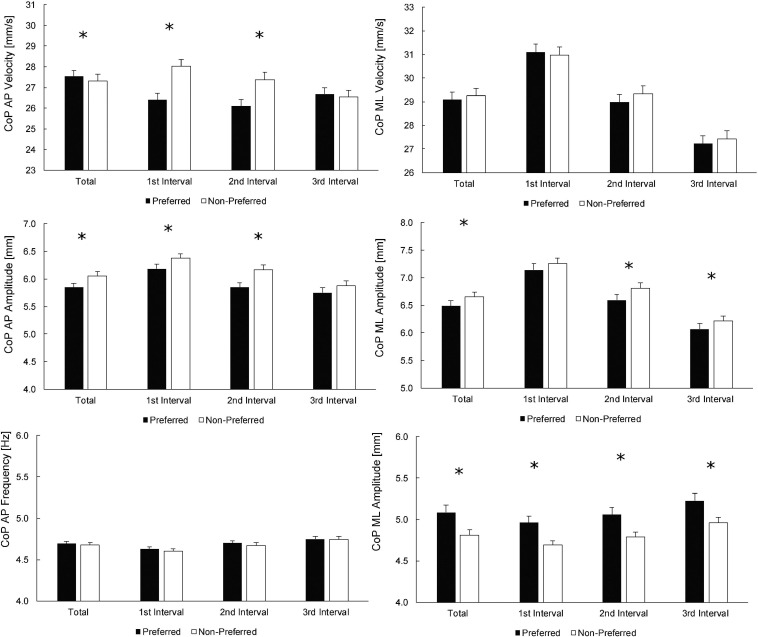
Comparisons between the preferred and non-preferred legs (according to the classification related to handedness). Statistically significant differences between the legs for whole-trial (total; leftmost columns) values, as well as for individual time intervals, are denoted by asterisks. For main effects of leg and time intervals, see Table 2 (bottom half).

Figure 2 depicts the values for the preferred and non-preferred legs, according to the handedness. Most interestingly, both CoP AP velocity and CoP AP amplitude showed statistically significant differences between the legs for the whole trial and for the 1st and 2nd intervals, but not the 3rd interval. In particular, the results imply that the preferred leg exhibited superior balance only during the 1st and 2nd intervals of the trial, but not the 3rd interval, which probably explains leg × time interaction for these two parameters (see Table 2 for details). On the contrary, CoP ML amplitude was only different between the legs at the 2nd and 3rd intervals, but not at the 1st interval, which is again reflected in statistically significant leg × time interaction from ANOVA. All the effect sizes for differences between the legs were small (Cohen’s *d* = 0.05–0.14). The effects of the leg preference on CoP AP frequency were not found (*p* ≥ 0.475), whereas CoP ML frequency was consistently higher in the preferred leg (all *p* < 0.001).

Additional comparisons between the preferred and non-preferred legs were done for DIF_21 and DIF_31 (i.e., the relative difference between the 1st and the 2nd and the 1st and the 3rd 10 s intervals). None of these outcome variables exhibited statistically significant differences between the legs (*p* = 0.102–0.904), when the preferred leg was determined based on single-leg jumping preference (Table 3, upper half). On the other hand, as shown above by ANOVA, the whole-trial estimates did exhibit statistically significant differences between the legs, although the effect sizes were very small (η^2^ = 0.01–0.02).

**TABLE 3:** Transient characteristics of body sway in the preferred and non-preferred legs.

Preference—jumping	Preferred		Non-preferred		Difference		
	Mean	SD	Mean	SD	*T*	*P*	ES
CoP velocity—total (DIF_21)	96.73	11.64	96.38	11.21	0.63	0.530	0.03
CoP velocity—total (DIF_31)	93.53	15.29	92.38	12.84	1.64	0.102	0.08
CoP velocity—AP (DIF_21)	98.44	14.26	97.94	12.63	0.76	0.451	0.04
CoP velocity—AP (DIF_31)	97.27	20.24	95.85	14.47	1.57	0.116	0.08
CoP velocity—ML (DIF_21)	95.76	11.87	95.56	12.49	0.32	0.746	0.02
CoP velocity—ML (DIF_31)	90.51	13.13	89.80	14.46	1.08	0.281	0.05
CoP amplitude—AP (DIF_21)	99.19	20.28	99.30	18.80	−0.12	0.904	0.01
CoP amplitude—AP (DIF_31)	97.46	31.01	95.95	19.68	1.12	0.263	0.06
CoP amplitude—ML (DIF_21)	97.01	20.01	97.68	21.43	−0.67	0.501	0.03
CoP amplitude—ML (DIF_31)	89.42	20.02	90.10	35.00	−0.47	0.642	0.02
CoP frequency—AP (DIF_21)	103.32	10.11	102.37	9.46	1.83	0.068	0.10
CoP frequency—AP (DIF_31)	104.65	11.33	103.81	10.15	1.53	0.128	0.08
CoP frequency—ML (DIF_21)	103.71	12.24	102.76	11.56	1.63	0.104	0.08
CoP frequency—ML (DIF_31)	106.84	13.16	106.33	14.14	0.75	0.456	0.04

**Preference—handedness**	**Preferred**		**Non-preferred**		**Difference**		
	**Mean**	**SD**	**Mean**	**SD**	***T***	***P***	**ES**

CoP velocity—total (DIF_21)	95.75	11.01	97.36	11.75	−2.96	0.003	0.14
CoP velocity—total (DIF_31)	92.86	15.66	93.04	12.39	−0.25	0.803	0.01
CoP velocity—AP (DIF_21)	97.30	12.54	99.06	14.27	−2.71	0.007	0.13
CoP velocity—AP (DIF_31)	96.57	19.84	96.53	15.04	0.05	0.958	0.00
CoP velocity—ML (DIF_21)	94.99	12.38	96.35	11.94	−2.31	0.021	0.11
CoP velocity—ML (DIF_31)	89.99	14.82	90.33	12.71	−0.51	0.611	0.02
CoP amplitude—AP (DIF_21)	98.09	18.06	100.39	20.87	−2.35	0.019	0.12
CoP amplitude—AP (DIF_31)	97.06	30.10	96.33	21.03	0.54	0.587	0.03
CoP amplitude—ML (DIF_21)	96.76	21.05	97.98	20.40	−1.22	0.223	0.06
CoP amplitude—ML (DIF_31)	90.43	35.34	89.12	19.36	0.90	0.369	0.05
CoP frequency—AP (DIF_21)	102.91	9.58	102.77	10.05	0.27	0.790	0.01
CoP frequency—AP (DIF_31)	104.04	10.83	104.43	10.69	−0.70	0.483	0.04
CoP frequency—ML (DIF_21)	103.17	12.10	103.26	11.73	−0.16	0.876	0.01
CoP frequency—ML (DIF_31)	106.34	14.46	106.84	12.79	−0.74	0.461	0.04

*CoP, center-of-pressure; AP, anterior–posterior; ML, medial–lateral; DIF_21, relative difference between the 1st and 2nd intervals within the whole trial; DIF_31, relative difference between the 1st and 3rd intervals within the whole trial; ES, effect size (Cohen’s d). DIF_21 and DIF_21 are expressed as percentages (100% representing no change;>100% indicating an increase in time;<100% indicating a decrease in time).*

There was statistically significant difference in DIF_21 between the legs when the preference was determined based on handedness for CoP total velocity, CoP AP velocity, CoP ML velocity, and CoP AP amplitude (*p* = 0.003–0.021); however, the effect sizes were small (Cohen’s *d* = 0.11–0.14) (Table 3, bottom half). The same four variables showed statistically significant leg × time interval interactions (see Table 2, bottom half).

In line with the statistically significant main effect of time interval from ANOVA (for both approaches to leg preference classification), a decrease in CoP velocity and amplitude variables throughout the trial was reflected in DIF_21 and DIF_31 below 100% value, whereas the increase in the CoP frequency variables was reflected in DIF_21 and DIF_31 above 100%.

Across all parameters (whole trials, individual intervals, and DIF_21/DIF_31), we also calculated the percentage of participants for which the preferred leg showed better balance performance (for the purposes of these analyses, it was assumed that higher CoP frequency indicates superior balance). Regardless of the approach for limb classification, the range of percentage values was 42–58%, which indicates that, in addition to small effects, there was no clear consistency for the preferred or non-preferred leg to exhibit better balance performance.

## Discussion

The purpose of this paper was to analyze transient characteristics of body sway in healthy athletic population and to explore the effects of leg preference on single-leg standing body sway variables. Firstly, it was found that the transient characteristics of the body sway (expressed as relative differences between individual time intervals within the trial; i.e., DIF_21 and DIF_31) were not associated or weakly associated with the whole-trial estimates of corresponding variables. All body sway variables showed transient characteristics, reflected in decrease (CoP velocity and amplitude) or increase (CoP frequency) throughout the trial. When the preferred leg was determined based on the participants’ preference for single-leg jumping, very small effects of the leg were observed for CoP amplitude and CoP velocities, with the non-preferred leg showing smaller values, though the differences were much smaller than the differences between individual time intervals within the trial. No leg × time interval interactions were observed, and no differences between the legs were noted for DIF_21 or DIF_31. On the other hand, when the preferred leg was based on the handedness (i.e., the preferred leg matching the side of the preferred hand for writing and eating), leg × time interval interactions were observed for all CoP velocity variables and CoP AP amplitude, and consequently, statistically significant differences were noted between the legs in transient characteristics (in all cases for DIF_31). In terms of CoP AP velocity and CoP AP amplitude, it appears that the preferred leg showed better balance (lower values) only during the 1st and 2nd intervals, but not the 3rd interval of the whole trial. In contrasts, the legs showed similar CoP ML amplitude during the 1st interval, whereas the preferred leg showed lower values in the 2nd and 3rd intervals.

Our first hypothesis was confirmed, as the correlations between whole-trial estimates and corresponding DIF_21 and DIF_31 were negligible or small. This is in accordance with Reed et al. (2020), who used parallel stance task and showed similar correlation coefficients (−0.12 > *r* > 0.21). Certain variables in our study showed statistically significant small positive correlations (notably for CoP velocity and amplitude). This would imply that individuals with larger and faster CoP movement also exhibit larger DIF_21 and DIF_31, which means that they also stabilize less throughout the trial (note that for DIF_21 and DIF_31, a value of 100% represents no change through time intervals; >100% indicates an increase in time; <100% indicates a decrease in time). Previous studies have indicated that transient characteristics of body sway or time interval-specific values could offer additional insights into individual’s postural control (Boucher et al., 1995; Brown et al., 2006; Reed et al., 2020); it remains open to further investigations to explore if this approach to body sway analysis provides any additional practical and clinical utility. This could be explored by performing new experiments or possibly by re-analyzing existing datasets, as also suggested by Reed et al. (2020). For instance, several previous studies using instrumented body sway analyses or non-instrumented balance test were typically able to show statistically significant difference between fallers and non-fallers in the population of older adults (Kozinc et al., 2020). On the other hand, the specificity and sensitivity of such test for prediction of falls were moderate at best. It could be that transient characteristics of body sway could represent important additional information and/or clinical relevance related to individual’s postural control. Therefore, we encourage the researchers to include the analysis of transient characteristics in future studies and to re-analyze their existing datasets if possible.

In this paper, we additionally explored a more fundamental aspect of body sway and its transient characteristics, in particular the effects of limb preference. Several previous studies have investigated the differences between the limbs in terms of postural balance (Hoffman et al., 1998; Matsuda et al., 2008; Barone, 2010; Alonso et al., 2011; Muehlbauer et al., 2014; Promsri et al., 2018); however, statistically significant results were rarely observed (Barone, 2010; Promsri et al., 2018). In terms of the upper limb, it is well recognized that each arm/hand is specialized for specific control processes, which is related to functional hemispheric asymmetries (Zijdewind et al., 1990; Sainburg, 2005). The limb that is typically determined as the preferred limb is specialized for controlling trajectory dynamics, whereas the opposite limb is specialized for controlling position (Sainburg, 2005). Such specialization was also implied for lower limbs by functional magnetic resonance studies (Kapreli et al., 2006); however, the effects of functional hemispheric asymmetries on single-leg postural control are unexplored. Notably, it was also shown that the side of the preferred upper limb is almost exclusively (in 96% of cases) matched with the side of the preferred leg for kicking movements (Promsri et al., 2018). If the specialization for specific control processes in the lower limbs would reflect the one recognized in the upper limbs (Sainburg, 2005), it could be expected that the non-preferred limb would exhibit better postural control during static tasks, reflected in lower body sway. However, the opposite effects were found in our study, whereas previous studies either showed no differences between the limbs (Hoffman et al., 1998; Matsuda et al., 2008; Alonso et al., 2011; Muehlbauer et al., 2014) or, similarly to our results, showed better postural control of the preferred limb (Barone, 2010; Promsri et al., 2018). In view of their results, Promsri et al. (2018) suggested that single-leg standing could be seen as dynamic, rather than static task. Regardless if the hemispheric asymmetry produces differences in the control of movements in the lower limb or not, the effect of leg preference on body sway is probably small at best. When the preferred leg was determined as “the leg you would use for single-leg jumps to achieve maximal jump height,” the opposite effect was seen in our study, with the preferred leg showing higher body sway, and the effect sizes were similarly small.

When the leg preference was determined based on handedness, our analyses also showed interactions between the leg and time intervals, and related to that, differences between the legs in transient characteristics of body sway (DIF_31 in all cases). Specifically, it appears that CoP AP velocity and CoP AP amplitude are lower for the preferred leg during the 1st and 2nd intervals, but similar between the legs for the 3rd interval of the whole trial. On the other hand, the legs showed similar CoP ML amplitude during the 1st interval, whereas the preferred leg showed lower values in the 2nd and 3rd intervals. This suggests that in AP direction, the preferred leg was more stable (i.e., showing lower CoP amplitude and velocity) from the beginning of the trial, whereas the non-preferred leg “caught-up” only within the last interval. In the ML direction, the legs started off with similar amplitude; however, the sway of the preferred leg decreased more throughout the trial. In summary, the results suggest superior ability of the preferred leg, reflected in quicker stabilization (within the 1st interval) in AP direction and larger decrease in body sway throughout the trial in ML direction. It could be speculated that the differences arise from better ability of sensory reweighing (Peterka, 2018). However, the transition from double- to single-leg stance induces only a change in the somatosensory information inflow, whereas the visual and vestibular systems are unperturbed. Future studies are needed to explore the transient characteristics of body sway after removal or perturbation of sensory information from one or more of the systems, which would be expected to cause larger initial destabilization and more pronounced transient behavior of body sway (Reed et al., 2020). Compared with small effects of leg preference, larger effects will likely be observed when comparing different populations or subgroups (Boucher et al., 1995; Brown et al., 2006; Reed et al., 2020). Indeed, Reed et al. (2020) reported statistically significant differences between young and older participants regarding the transient characteristics of body sway. However, they did not report the differences between groups in whole-trial estimates. Therefore, it remains unknown whether the transient characteristics of body sway are less, similarly, or more sensitive to aging-related changes than whole-trial estimates. Moreover, it remains open for further studies to explore how the transient characteristics are related to risk of falling and performance of activities of daily life. Intuitively, appropriate immediate postural responses are more important when an individual encounters sudden external perturbation. Nonetheless, the ability of sensory reweighing could also be clinically relevant for certain real-life situations (e.g., turning-off the lights, transition to less stable surface, transitions to single-leg stance, etc.). Finally, future studies should investigate how the transient characteristics are affected by different conditions and interventions, such as different plantar inserts (Tramontano et al., 2019).

Some limitations of the study need to be acknowledged. The body sway assessment was performed during a single-visit cross-sectional experiment, conducted within a larger study. The extended protocol also involved the assessment of passive range of motion, isometric lower limb strength, and vertical jumping ability. Therefore, a certain level of fatigue cannot be ruled out, although participants were provided with sufficient brakes. Moreover, the sample used within this study was highly heterogeneous, as also by statistically significant group effect for all CoP variables. However, because leg × time interval × group, as well as leg × group and time interval × group interactions, was all statistically non-significant, we further treated all the participants as one group. Nevertheless, a certain level of confounding effects due to sample heterogeneity cannot be ruled out. Finally, a considerable (CV > 10%) within-participant variance between the repetitions was found for CoP amplitude variables for individual time intervals within the trial and for DIF_21 and DIF_31. Indeed, averaging the CoP data across trials of longer duration (≥30 s) is known to increase the reliability of the body sway measurements (Reed et al., 2020). However, the whole-trial estimates (i.e., averaging the CoP data across the trial) may mask transient postural characteristics. While the assessment of transient characteristics seems to be promising, caution is needed because of potentially lower reliability. In particular, future researchers should be cautious about the reliability of posturographic assessments and the choice of outcome variables when investigating different clinical populations (Tamburella et al., 2014). Future studies should be conducted to assess intra- and inter-session reliability of the transient characteristics of body sway. Finally, it has to be stressed that the present findings cannot be extrapolated to the dynamic tasks (e.g., a continuous dynamic task with high demand for stability), as the role and contribution of individual sensory systems, as well as the process of sensory integration, is likely different in dynamic compared with static tasks (Bent et al., 2002, 2005). More studies will also be needed to better clarify the role of each sensory system. Because this study assesses the body sway only in the open eyes condition, it remains unknown if the transient characteristics of the body sway would be different in eyes closed condition or with the addition of other sensory manipulation.

## Conclusion

This study confirmed that the transient characteristics of the body sway are not associated with whole-trial estimates of corresponding variables. Moreover, all the body sway variables showed transient characteristics. Further studies should focus on examining the clinical relevance of transient characteristics of body sway, such as sensitivity to aging-related changes and risk of falling. The differences between the preferred and non-preferred legs were very small, in terms of both whole-trial estimates and transient characteristics. Notably, it was indicated that the preferred leg could exhibit superior postural control, as it appeared that it was able to reduce the body sway earlier and to a greater extent during the 30 s single-leg stance trial.

## Data Availability Statement

The raw data supporting the conclusions of this article will be made available by the authors, without undue reservation.

## Ethics Statement

The studies involving human participants were reviewed and approved by Republic of Slovenia’s National Medical Ethics Committee. Written informed consent to participate in this study was provided by the participants’ legal guardian/next of kin.

## Author Contributions

ŽK and NŠ conceptualized the idea. NŠ overviewed the measurement procedures and administration. ŽK analyzed the collected data and wrote the manuscript. NS and ŽK finalized the manuscript. Both authors contributed to the article and approved the submitted version.

## Conflict of Interest

NŠ was employed by a company S2P, Science to Practice, Ltd., Ljubljana, Slovenia. The remaining author declares that the research was conducted in the absence of any commercial or financial relationships that could be construed as a potential conflict of interest.
